# Transforming community prevention systems for sustained impact: embedding active implementation and scaling functions

**DOI:** 10.1007/s13142-015-0351-y

**Published:** 2016-02-03

**Authors:** William A. Aldridge, Renée I. Boothroyd, W. Oscar Fleming, Karen Lofts Jarboe, Jane Morrow, Gail F. Ritchie, Joyce Sebian

**Affiliations:** FPG Child Development Institute, University of North Carolina at Chapel Hill, CB #8185, Chapel Hill, NC 27599-8185 USA; Child and Family Policy Institute of California, Sacramento, CA USA; The North Carolina Partnership for Children, Raleigh, NC USA; Substance Abuse and Mental Health Services Administration, Center for Mental Health Services, Rockville, MD USA; Smart Start of New Hanover County, Wilmington, NC USA

**Keywords:** Community prevention, Evidence-based practice, Implementation, Systems change, Scaling, Infrastructure, Type 4 translational research

## Abstract

Traditional efforts to translate evidence-based prevention strategies to communities, at scale, have not often produced socially significant outcomes or the local capacity needed to sustain them. A key gap in many efforts is the transformation of community prevention systems to support and sustain local infrastructure for the active implementation, scaling, and continuous improvement of effective prevention strategies. In this paper, we discuss (1) the emergence of applied implementation science as an important type 3–5 translational extension of traditional type 2 translational prevention science, (2) active implementation and scaling functions to support the full and effective use of evidence-based prevention strategies in practice, (3) the organization and alignment of local infrastructure to embed active implementation and scaling functions within community prevention systems, and (4) policy and practice implications for greater social impact and sustainable use of effective prevention strategies.

## INTRODUCTION

The translational model of prevention science introduced in this special issue offers a novel and important step forward by incorporating the implementation and scaling of rigorously tested prevention strategies within local, state, national, and global prevention systems (see types 3–5 translation, Table 1). The ultimate success of a translational process cannot be measured by the effectiveness of prevention programs, practices, and policies alone but must also include the ability to bring the full experience of effective prevention strategies to children, families, and communities and achieve intended well-being outcomes at scale. Making the later stages of the translational process complex is that community prevention systems are often sizable, loosely structured configurations of community service organizations and public service agencies. These organizations and agencies employ a workforce with diverse types and levels of training; mix public and private, for-profit and not-for-profit organizational structures with diverse funding sources; and are regulated by an array of different local, state, and federal policies. In many communities, the prevention system may lack formal recognition or visibility. The effective implementation and scaling of prevention science in our communities and the realization of intended benefits have been generally elusive outside of pockets of excellence with unique conditions that are often difficult to replicate (e.g., unusual financial, service system, and human resource factors).

**Table 1:** Translational research stages

Type	Type 0 translation (T0)	Type 1 translation (T1)	Type 2 translation (T2)
Definition	The fundamental process of translating findings and discoveries from social, behavioral and biomedical sciences into research applied to prevention intervention.	Moving from bench to bedside. Translation of applied theory to methods and program development.	Moving from bedside to practice and involves translation of program development to implementation.
Type	Type 3 translation (T3)	Type 4 translation (T4)	Type 5 translation (T5)
Definition	Determining whether efficacy and effectiveness trial outcomes can be replicated under real world settings.	Wide-scale implementation, adoption and institutionalization of new guidelines, practices, and policies.	Translation to global communities. Involves fundamental, universal change in attitudes, policies, and social systems.

Prevention science is not alone in this difficulty. The literature on adopting evidence-based human services interventions has shown that outcomes are not routinely reproduced in real-world service settings [1–4]. Where effective innovations are adopted, disparate outcomes between research trials and real-world use are often due to implementation concerns: difficulties supporting delivery of the innovation(s) as intended or at scale within naturally occurring service environments [5–11]. Effective implementation has been identified as an essential yet often neglected component of the science-to-service translation process [6, 12–14]. Far too often, science-to-service translation has relied only on the processes of diffusion (i.e., the passive spread of intervention knowledge) or dissemination (i.e., the persuasion of a group to adopt an intervention with the compliment of basic training programs and materials) [15, 16]. These processes are often insufficient to bring about sustainable support for innovative strategies within long-standing, complex service systems. Furthermore, they place accountability for the consistent, intended delivery of innovative practices largely on practitioners with only the limited knowledge, skills, and abilities they may acquire from the diffusion or dissemination process. In contrast, creating meaningful and lasting change involves dynamic strategies to transform complex service systems into suitable host environments for effective prevention strategies [15, 17–19]. Successful and sustainable implementation of effective prevention strategies, and the achievement of expected well-being outcomes at scale, require the alignment of a supportive network of community prevention organizations that transfer accountability from practitioners to the community prevention system as a whole.

## APPLIED IMPLEMENTATION SCIENCE

Given the lack of success with traditional diffusion and dissemination processes, over the past decade, applied implementation science has started to coalesce around core *active implementation* processes [12, 14, 15, 20, 21]. This focus has largely emerged from the identification of processes associated with more effective implementation and greater realization of expected outcomes [6, 13]. Synthesizing the extant literature in applied implementation science and the experiences of a broad range of stakeholders, the National Implementation Research Network developed five Active Implementation Frameworks to describe and guide the process of actively implementing evidence-based interventions at scale in typical human service settings. The frameworks—Usable Interventions, Implementation Teams, Implementation Drivers, Implementation Stages, and Improvement Cycles—are well detailed by Fixsen and colleagues [6, 13, 15] and Metz and Bartley [20]. The Active Implementation Frameworks offer essential concepts and strategies for effectively moving type 2 translational prevention science into practice within real-world community prevention systems.

## ACTIVE IMPLEMENTATION AND SCALING FUNCTIONS

**Fig. 1:**
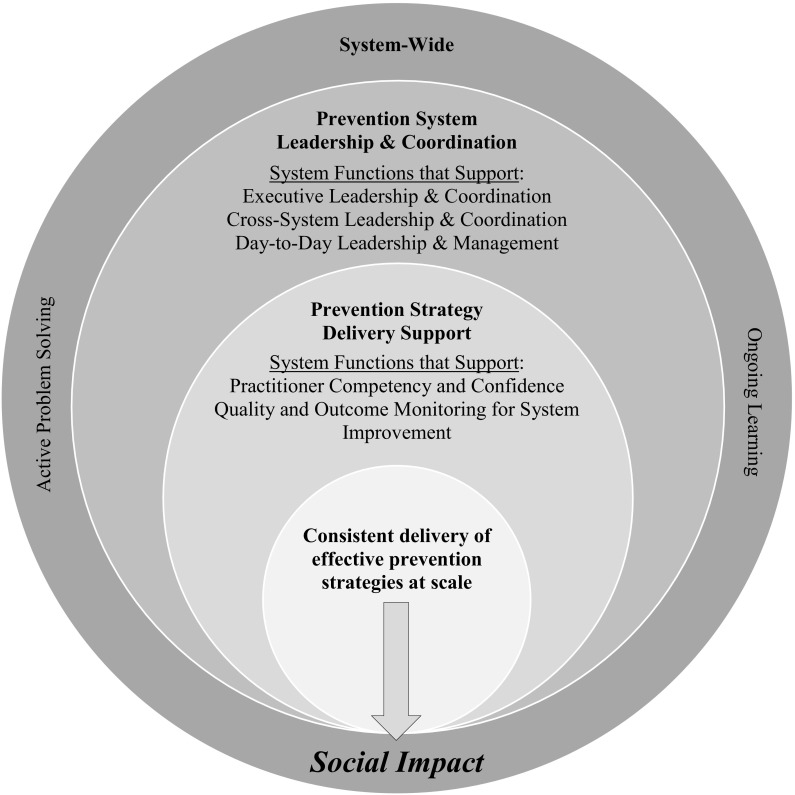
Nesting of the active implementation and scaling functions within community prevention systems to achieve social impact

**Fig. 2:**
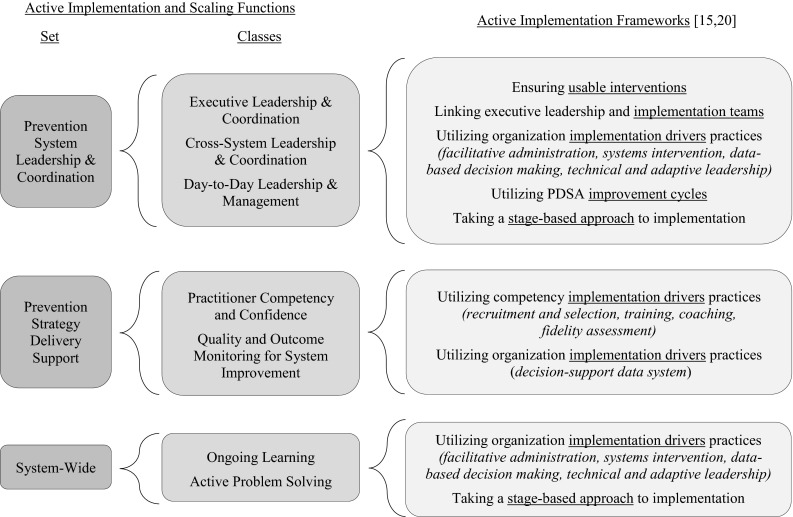
Crosswalk of the active implementation and scaling functions and the Active Implementation Frameworks

Applying the science of active implementation throughout complex systems environments can be challenging. However, when successful, our experience is that the application of the concepts and strategies within the Active Implementation Frameworks results in an array of coordinated system functions being embedded in community prevention systems to actively support implementation and scaling. As illustrated in Fig. 1, these *active implementation and scaling functions*, organized within three sets and detailed in Table 2, nest within and rely on each other. Together, they create a nurturing environment for the consistent delivery of effective prevention strategies to achieve social impact. Furthermore, early consideration of these functions allows community leaders and partners, policymakers, and funders a way to consider function before committing to form in transforming their community prevention system to successfully host effective prevention strategies—whether individually or in multicomponent packages. For example, implementation teams with clear roles and responsibilities can be created or repurposed around the functions while attending to existing features of the community prevention system, such as size, history, resources, culture, and political and social complexities. As they better understand these functions and their integration, community leaders and partners are also able to more effectively organize and align cross-system implementation infrastructure, such as recruitment and selection, training, coaching, fidelity assessment, and data collection and monitoring systems. To facilitate understanding of how the active implementation and scaling functions emerge from application of the Active Implementation Frameworks, a crosswalk is provided in Fig. 2.

**Table 2:** Active implementation and scaling functions within a community prevention delivery system

Prevention system leadership & coordination
Executive
1. Demonstrate ongoing commitment to the implementation and scaling of community prevention strategies to achieve intended outcomes for community youth and families.
2. Demonstrate ongoing commitment to community partnerships to ensure that multicultural values and experiences are incorporated into practice and system changes.
3. Create appropriate opportunities for change within the community prevention delivery system.
4. Nurture systems change once it is underway.
Cross-system
1. Select community prevention strategies to respond to identified community needs.
2. Align community prevention strategies under a common approach to implementation.
3. Select and align community prevention delivery agencies to attain community-wide reach.
4. Review and recommend solutions to shared implementation barriers and system needs, incorporating the perspectives of key prevention system and community partners.
5. Facilitate and normalize communication about systems changes and successes among and across all stakeholders and community members.
Day-to-day
1. Ensure that community prevention strategies are teachable, learnable, doable, and assessable in practice.
2. Assess and create ongoing “buy-in” and readiness across the community prevention delivery system.
3. Install, ensure the aligned operation of, and sustain implementation infrastructure and best practices.
4. Develop and implement action plans to manage stage-based work.
5. Ensure the use of data, including fidelity and outcome data, across the community prevention system for continuous improvement.
6. Involve key prevention system and community partners, including youth and families, in implementation activities and decision-making for system improvement.
7. Organize and direct the day-to-day flow of information to support implementation.
8. Identify and address implementation barriers and ensure the spread of solutions to support successful implementation.
Prevention strategy delivery support
Practitioner competency and confidence
1. Select practitioners who demonstrate alignment with the philosophy, values, and principles of chosen community prevention strategies.
2. Develop practitioners’ initial knowledge, skills, and abilities to deliver chosen community prevention strategies as intended.
3. Improve practitioner’s ongoing ability to effectively deliver community prevention strategies across diverse families and contexts.
Quality and outcome monitoring for system improvement
1. Assess whether the core components of the community prevention strategies are consistently being delivered as intended.
2. Gather, manage, and report data about community prevention strategies and their implementation to inform ongoing decision-making and continuous quality improvement.
System-wide
Ongoing learning
1. Prioritize learning for continuous improvement.
2. Value community youth and families’ preferences and experiences.
3. Use data to make decisions.
4. Take time to identify and build readiness for the next right steps.
Active problem solving
1. Identify local administrative and service delivery needs and respond with facilitative solutions.
2. Identify prevention system needs and advocate for appropriate solutions with system partners.
3. Use appropriate technical and adaptive strategies to respond to prevention system and service delivery challenges.
4. Communicate purposefully and regularly to nurture engagement across the community prevention system.

### Prevention system leadership and coordination

The first set of active implementation and scaling functions, collectively organized under “prevention system leadership and coordination” in Table 2, incorporates many elements of a public health approach to prevention and social inequity reduction, such as operating under shared vision and principles, active community involvement, prioritizing and advancing chosen prevention strategies through diverse access points, and the utilization of ongoing assessment and data driven decision-making [17, 22]. Given the size and often loose structure of community prevention systems, the successful implementation and scaling of effective prevention strategies require the alignment of collaborative organizations with coordinated leadership and accountability. The scope of community-wide prevention also requires considerable support and management on a day-to-day basis. Thus, within prevention system leadership and coordination, there are three classes of functions: executive, cross-system, and day-to-day.

#### Executive leadership and coordination

The active implementation and scaling functions for executive leaders within the community’s prevention system ensure an ongoing commitment that chosen prevention strategies will be effectively and sustainably supported. Those with executive leadership often have the power to re-direct resources, modify staff position descriptions, change the way community organizations work together, raise awareness and support for transformational change within a community, and create an active, involved partnership with community youth and families. By demonstrating a sustained commitment to the implementation and scaling of chosen prevention strategies, incorporating diverse cultural values and experiences from the community, and creating and nurturing the systems changes needed to support chosen prevention strategies, executive leaders across community sectors create a hospitable climate for innovation [15, 23, 24]. The climate that is fostered by these leaders will go a long way to ensuring the success and sustainability of chosen community prevention strategies or allowing support for chosen strategies to drift or collapse over time [25–27].

#### Cross-system leadership and coordination

Cross-system active implementation and scaling functions ensure the alignment of prevention system activities, stakeholders, and community members. Prevention strategies must be chosen to respond to identified community well-being needs and to work in harmony under often limited resources. Prevention delivery agencies need to be recruited into community prevention coalitions under a common vision and strategic plan for the implementation and scaling of chosen prevention strategies and to create optimal reach into the community via diverse access points (e.g., education, mental health, primary care, and media). These agencies can benefit from shared implementation infrastructure for chosen prevention strategies, particularly related to the training and coaching of practitioners, fidelity assessment, ongoing data collection and analysis, media, and system leadership and technical support. By sharing implementation infrastructure, common implementation barriers and system needs can be identified, leading to greater system efficiencies as solutions spread across partner agencies [15]. Finally, those ensuring cross-system implementation functions are tasked with facilitating and normalizing communication about prevention system practice and policy changes to create awareness for the next steps ahead and share successes [15]. Cross-system leadership and coordination functions have been captured within structures such as Communities That Care community prevention boards [28] and Community Development Teams, originally developed by the California Institute of Mental Health and later used to support the implementation of Multidimensional Treatment Foster Care in California [29, 30].

#### Day-to-day leadership and management

Often, the most intensive work occurs in attending to day-to-day active implementation and scaling functions. These functions require close, ongoing leadership and management from dedicated implementation team members serving the community prevention system. These teams are tasked with ensuring that chosen prevention strategies are usable [31]; creating readiness for new behaviors among system stakeholders; actively involving community partners; and installing, ensuring the aligned operation of, and sustaining implementation infrastructure to support chosen strategies [15]. This work entails strong organizational and system development skills including using data for setting priorities and continuous quality improvement, engaging in active problem solving, and developing trusted relationships with diverse stakeholders with sometimes competing agendas [24, 32, 33]. Moreover, managing these functions requires commitment to a shared community vision, a tolerance for ambiguity and slow pace, risk-taking, and the ability to create and manage action plans in accordance with identified stages of implementation [15, 20, 32–34]. In North Carolina, several counties scaling the Triple P—Positive Parenting Program—system of interventions are attending to these day-to-day functions using teams of county public health managers with the collective skills and expertise to actively drive implementation across coalitions of public agencies, pediatric clinics, schools, and other community organizations.

### Prevention strategy delivery support

Represented in Fig. 1, the prevention system leadership and coordination functions provide a nurturing environment for the second set of active implementation and scaling functions, collectively organized under “prevention strategy delivery support” in Table 2. This second set of system functions, grouped into two classes, directly supports the consistent and effective delivery of prevention strategies to achieve social impact.

#### Practitioner competency and confidence

The first class of system functions ensures the competency and confidence of practitioners to deliver chosen prevention strategies as intended and with expected benefits to community youth and families. To start, practitioners with sufficient capacity must be recruited or selected to deliver chosen prevention strategies to create community-wide reach. Having a priori selection criteria that go beyond general qualifications and experiences to evaluate the fit of candidates with the philosophy, values, and principles of chosen prevention strategies may greatly facilitate practitioner effectiveness with chosen strategies, reduce resistance to change in the community prevention system, and prevent practitioner burnout and turnover. Although individual community agencies often play a strong role in the selection of their practitioners to participate in new programs, community prevention system leaders can successfully advocate for the cross-system use of selection best practices and selection criteria that are aligned with adopted prevention strategies.

Next, selected practitioners need effective training, using adult learning best practices [35], to deliver chosen prevention strategies. Trainings can be well done when outsourced to a qualified prevention strategy developer or purveyor, although local plans are often needed to align resources and timelines for training large numbers of practitioners across multiple agencies and prevention strategies. On the other hand, some prevention strategy developers and purveyors afford the opportunity to develop local training capacity through trainer certification processes. These opportunities may have long-term benefits but also require additional considerations, such as the selection, training, and ongoing quality assurance of locally certified trainers.

After training, in-service coaching of practitioners is essential to increase practitioners’ use of new strategies [36] and for improving their effectiveness across diverse youth and families [37–39]. Ongoing coaching of practitioners may be accomplished through cross-system coaching sessions, integrating coaching for new prevention strategies within existing agency supervision practices, or some combination. Regardless, coaching service delivery plans can be developed and implemented to ensure that practitioners across the community prevention system receive regular and effective coaching.

#### Quality and outcome monitoring for system improvement

The second class of system functions that directly supports the delivery of prevention strategies involves quality and outcome monitoring for continuous improvement. To ensure the intended delivery and optimize the benefits of chosen prevention strategies within dynamic community and service system environments, implementation processes and prevention strategy outcomes must be continuously monitored and improved [24]. This starts with ongoing fidelity assessment to ensure that the core components of chosen prevention strategies [31] are consistently being delivered across community youth and families. Aarons and colleagues [40, 41] demonstrated that fidelity monitoring of evidence-based practices in the context of supportive coaching may not increase burden or burnout among practitioners and may actually increase practitioner retention over time. Furthermore, if program developers have established a link between core intervention components and outcomes, fidelity data allows outcomes to be appropriately interpreted: Favorable outcomes may be attributed to prevention strategy effectiveness (in the known presence of core intervention components) and unfavorable outcomes may be correctly attributed to either a problem with the strategy’s effectiveness (in the known presence of core intervention components) or an implementation problem (in the known absence of core intervention components) [31]. Across the community prevention system, practical and efficient fidelity assessments must be developed or adopted for each strategy. Additionally, system-wide policies for reporting fidelity data are needed to inform quality improvement efforts.

Additional implementation data, such as the effectiveness of practitioner training and coaching practices, the quality of the implementation climate set by system leadership, and the effectiveness of implementation support teams, can also be used to ensure high-quality and sustainable implementation. Youth and family outcome data must be shared across the community prevention system to ensure that socially meaningful benefits are being realized. This may require data sharing agreements that protect privacy and comply with federal and state guidelines. Community prevention systems also need to secure the human resources and information technology needed to support the ongoing collection, analysis, and use of data for local decision-making and system improvement.

### System-wide

All staff within a community prevention system, from front-line practitioners to executive leaders, share a responsibility to create and nurture a culture that supports the successful implementation and scaling of community prevention strategies. Such a culture is enabled by ongoing attention to several functions that are shared at all levels of the community prevention system. This last set of system functions, collectively organized under “system-wide” in Table 2 and depicted as the broader sphere of influence in Fig. 1, are also grouped into two classes.

#### Ongoing learning

First, all community prevention system staff must prioritize their own learning to continually improve those aspects of the system over which they have direct control or influence. Implementing innovations requires an element of risk-taking that can be mitigated by systematic learning and improvement [24], whether at the practitioner level when delivering a new prevention strategy to a community youth or family, at the managerial level when supporting new practice routines, or at the executive level when adjusting agency policies and resources. Across all staff, valuing community youth and family preferences and experiences and using available data must support ongoing learning and decision-making. Listening to diverse community voices and engaging as partners in implementation is necessary in order to collectively move forward with change. Furthermore, timely data can be powerful in making prevention system functioning more objective and transparent to enable continuous quality improvement. Finally, change is not incumbent in human systems [19]. Care must be taken to assess and prepare colleagues and partners for next steps in the implementation and delivery of new prevention strategies.

#### Active problem solving

All staff within a community prevention system must also share responsibility for local problem solving and regular, purposeful system communication. Everyone within a community prevention system has local responsibilities and activities over which they have some control, such as practitioners’ scheduling of client services, and organizational or systems practices over which they have less influence, such as practitioners’ compliance with mandated service records. To support the successful implementation and scaling of innovative prevention practices, all system participants must be willing to develop and implement supportive solutions to local challenges over which they have direct control and communicate and advocate for supportive solutions to system needs over which they have little or no influence. These activities require staff to be leaders within their own corner of the community prevention system, including diagnosing the adaptive (i.e., unknown or complex) and technical (i.e., known or routine) elements of problems and responding with appropriate strategies [19]. When active problem solving and communication functions are carried out system-wide, they promote practice-policy communication cycles [15, 20] by which common implementation barriers and system needs are identified and the spread of solutions to support the successful and sustainable implementation of new prevention strategies is enabled.

## ORGANIZING AND ALIGNING INFRASTRUCTURE TO ENSURE ACTIVE IMPLEMENTATION AND SCALING FUNCTIONS

Given the diversity of community prevention agencies and contexts, finding the right individuals, teams, and processes to support the full array of active implementation and scaling functions can be an early and evolving challenge. However, nurturing a collaborative network of community organizations to deliver the chosen prevention strategies and share accountability for all system functions is essential to achieving large-scale impact. Clearly understanding the scope of the implementation and scaling initiative and the breadth and complexity of the community prevention system involved will help define the challenge and inform the work to be done. An early task in organizing and aligning system infrastructure may be to leverage agencies ready for the tough work ahead while fostering broader system readiness. Agency readiness may include commitment to the shared framework for community prevention, a pledge of staff time and agency resources, the willingness to adjust agency practices and policies to support collective needs and goals, and the commitment of executive leaders to nurture strong working relationships across the community prevention system. Before engaging in community-wide initiatives to implement and scale innovative prevention strategies, some community agencies may benefit from strengthening general organizational skills and abilities [42]. Based on their prior studies, Prochaska, Prochaska, and Levesque [43] suggested that only about 20 % of individuals within organizations may be ready to change. Thus, building readiness for change is likely to be an important and ongoing effort in any community prevention initiative.

### The co-creation process

Among those involved in the implementation and scaling of community prevention strategies, the organization and alignment of implementation infrastructure to successfully support community prevention strategies are processes of co-creation. As described by Metz and Albers [32], funders and policymakers; program developers and purveyors; and local agency leaders, stakeholders, and community members play collaborative roles in the creation of visible implementation infrastructure. While local agency leaders, staff, and community members may have the most intensive roles and ultimate ownership of community implementation infrastructure, strong program purveyors or intermediaries [6] and the active commitments of federal, state, and/or local funders and policymakers to support and sustain community prevention strategies are essential. Purveyors and intermediaries facilitate the local usability of prevention strategies while supporting integrity in the delivery of core intervention components that are associated with expected outcomes. Funders and policymakers actively partner with communities to address system barriers and ensure sustainable resources. In addition to these partners, communities may benefit from external active implementation technical assistance as they shift from traditional methods of diffusion and dissemination to more purposeful and effective implementation strategies [15]. Each of these co-creation partners must sustain their involvement through full implementation of the chosen prevention strategies, although their roles may change in form or intensity over time. Furthermore, as a part of the community prevention system, each co-creation partner benefits themselves and the community from ongoing learning and active problem solving processes. Thus, they share responsibility for the system-wide functions described in Table 2.

### A robust exploration process

The exploration stage of implementation includes assessing the requirements for implementation and scaling, including system barriers that must be addressed [20]. During a robust exploration process, several questions guide the organization and alignment of implementation infrastructure. First, with the full array of active implementation and scaling functions in mind, partners in the co-creation process must ask and answer questions related to *who* and *where*. That is, *who* will be involved in managing and completing activities related to each group of system functions and *where* will accountability for each group of system functions live within the community prevention system? If a centralized team in the community prevention system ensures many of the functions, that team must have the capacity to be responsible for those functions across all agencies in the prevention system. If agencies ensure some functions locally, partners must ensure that agency teams supporting such functions remain aligned with and accountable to the overall prevention system. It is also not uncommon for activities related to some functions to be shared across multiple levels of a community prevention system. For example, a centralized implementation team may coordinate and schedule training events for new community prevention strategies, a program purveyor may deliver the training, and service agencies may allocate time and reimburse transportation for practitioners to attend trainings. Building a common understanding of and responsibility for these roles may challenge established ways of work, but linking multiple levels of support can ensure that the right partners are available to address emergent barriers in a timely way. Leadership and implementation team structures [15, 20, 44] with clear articles of organization and purpose, such as terms of reference or memorandums of agreement, and cross-system communication protocols that outline expectations about the frequency and purpose of communication may facilitate such understanding [15].

In addition to formalizing answers to questions about who, where, and how to share, partners must also define *plans to carry out activities* related to the prevention strategy delivery support functions. Plans for ensuring the recruitment and selection, training, and coaching of practitioners will need to be developed and documented, as well as plans for the collection, sharing, reporting, and use of data to monitor and continuously improve implementation processes and youth and family well-being outcomes. Together with articles of organization for system leadership and implementation teams, these plans will help answer questions about the *resources* that will be needed to support the implementation, scaling, and delivery of chosen prevention strategies. In addition, during the development of such articles and plans, it is usual for *complex, adaptive challenges* to be identified, such as how to remove practitioners from the front lines to attend training and coaching meetings or how agencies may participate in shared data collection and reporting. Such challenges have no easy solution [19, 45], and strategies to manage each challenge must be developed and implemented.

Finally, during the exploration stage of implementation, the early and active involvement of community members, including youth and families who will be served, and front-line agency staff is essential. Community members and agency practitioners are positioned at the nexus of the implementation process, and they often have unique perspectives on system needs and gaps that may prevent the high-quality delivery and sustainability of community prevention strategies [24]. They may also be able to identify complex issues that are more difficult for system or agency leaders to see, such as how families may be unintentionally impacted by certain community prevention strategies. When community members and practitioners are involved early, they can become advocates for chosen community prevention strategies and effectively organizing and aligning infrastructure to transform the community prevention system.

### Getting started with leadership and an implementation team

Given their extent and scope, many stakeholders struggle to get started with the process of organizing and aligning infrastructure to support the active implementation and scaling functions. The identification of a first-generation community implementation team [15, 20, 29, 44] is often an effective place to start. Effective implementation teams

- are comprised of at least three to five individuals with experience managing systems changes and data-based improvements to support the implementation of an innovation,
- incorporate expertise in chosen community prevention strategies as well as active implementation science and practice,
- are closely supported by and linked to executive leaders in the community prevention system, and
- are responsible for ensuring the cross-system and day-to-day leadership and coordination functions in Table 2 [15, 20].

Community implementation teams provide the mechanism by which accountability for high-quality evidence-based prevention services is intentionally transferred from practitioners alone to the community prevention system itself. Moreover, emerging evidence suggests that implementation teams may be able to reduce the time for evidence-based strategies to fully and effectively reach youth and families [29, 46] and increase sustainability of such strategies over time [46].

## CONCLUSIONS AND RECOMMENDATIONS

The gap between the processes needed to successfully and sustainably implement and scale effective prevention strategies within community prevention systems and the reality of what is occurring in many local communities poses a challenge for system stakeholders and the youth and families who can benefit from effective prevention. With a focus on closing that gap, applied implementation science and the Active Implementation Frameworks are an important type 3–5 translational extension of traditional type 2 translational prevention science. The active implementation and scaling functions found in Table 2, emerging from experiences applying the concepts and strategies within the Active Implementation Frameworks, acknowledge that many participants at every level of a community prevention system need to be highly engaged, committed, and accountable to make effective use of innovative prevention strategies. This challenge requires the collaborative support of funders and policymakers; program developers and purveyors; technical assistance providers; and local agency leaders, stakeholders, and community members to co-create visible implementation infrastructure for success and sustainability.

Policymakers at federal, state, and local levels and other funding entities can respond to the challenge of effective implementation and scaling through the use of funding requirements and program expectations. For example, a portion of allocated funding could be set aside for the development of local implementation infrastructure, such as leadership and implementation teams, as well as the development of local implementation processes, such as practitioner training and coaching protocols and fidelity assessment and continuous quality improvement systems. Additionally, policymakers and funders may employ funding strategies that allow for stage-based implementation activities (e.g., a planning year) and incorporate realistic periods to achieve full implementation and expected outcomes [34, 46–50]. Also important and challenging for states and communities is how they work together to support prevention. There is growing understanding that state–community partnerships are essential for implementing and scaling innovative prevention strategies. While cascading implementation teams and practice-policy communications cycles from the state capital through to community service agencies are essential elements of these partnerships [15, 20], additional research will be helpful in this area.

Other recommendations for research include the need for program developers to identify in published articles the core components of their prevention strategies so that community practitioners are clear about which strategy components directly affect intended outcomes and which ones might be flexibly adapted to local community context. Likewise, when reporting the outcomes of implementation trials, researchers would be wise to report fidelity data and the quality of active implementation and scaling infrastructure to support using prevention strategies as intended, as these may greatly influence strategy effectiveness and sustainability. Finally, through the establishment of partnerships reflecting trust and mutual self-interest [33, 51], prevention scientists and community stakeholders could increasingly come together to co-create implementation research questions from the lived experience of community preventionists. An area of study, for example, could be the co-creation process and how the expertise of each partner is incorporated and used effectively. Early indications may suggest that the quality of the interactions between co-creation partners is essential for a productive partnership [51].
